# Microbial Analysis of Saliva to Identify Oral Diseases Using a Point-of-Care Compatible qPCR Assay

**DOI:** 10.3390/jcm9092945

**Published:** 2020-09-11

**Authors:** Pune N. Paqué, Christopher Herz, Joël S. Jenzer, Daniel B. Wiedemeier, Thomas Attin, Nagihan Bostanci, Georgios N. Belibasakis, Kai Bao, Philipp Körner, Tanja Fritz, Julia Prinz, Patrick R. Schmidlin, Thomas Thurnheer, Florian J. Wegehaupt, Konstantinos Mitsakakis, Johannes R. Peham

**Affiliations:** 1 Clinic of Conservative and Preventive Dentistry, Center of Dental Medicine, University of Zurich, Plattenstrasse 11, 8032 Zurich, Switzerland; Joel.Jenzer@zzm.uzh.ch (J.S.J.); thomas.attin@zzm.uzh.ch (T.A.); philipp.koerner@zzm.uzh.ch (P.K.); Patrick.Schmidlin@zzm.uzh.ch (P.R.S.); thomas.thurnheer@zzm.uzh.ch (T.T.); florian.wegehaupt@zzm.uzh.ch (F.J.W.); 2 Austrian Institute of Technology, Molecular Diagnostics, Giefinggasse 4, 1210 Wien, Austria; christopher_herz@pall.com (C.H.); tanjafritz2@gmail.com (T.F.); Julia.prinz@usz.ch (J.P.); johannes.peham@ait.ac.at (J.R.P.); 3 Statistical Services, Center of Dental Medicine, University of Zurich, Plattenstrasse 11, 8032 Zurich, Switzerland; daniel.wiedemeier@zzm.uzh.ch; 4 Division of Oral Diseases, Department of Dental Medicine, Karolinska Institutet, 141 04 Huddinge, Sweden; nagihan.bostanci@ki.se (N.B.); george.belibasakis@ki.se (G.N.B.); kai.bao@ki.se (K.B.); 5 Hahn-Schickard, Georges-Koehler-Allee 103, 79110 Freiburg, Germany; Konstantinos.Mitsakakis@Hahn-Schickard.de; 6 Laboratory for MEMS Applications, IMTEK—Department of Microsystems Engineering, University of Freiburg, Georges-Koehler-Allee 103, 79110 Freiburg, Germany

**Keywords:** point of care, caries, periodontitis, oral health, saliva diagnostics, oral pathogens

## Abstract

Oral health is maintained by a healthy microbiome, which can be monitored by state-of-the art diagnostics. Therefore, this study evaluated the presence and quantity of ten oral disease-associated taxa (*P. gingivalis*, *T. forsythia*, *T. denticola*, *F. nucleatum, C. rectus*, *P. intermedia*, *A. actinomycetemcomitans*, *S. mutans*, *S. sobrinus*, oral associated *Lactobacilli*) in saliva and their clinical status association in 214 individuals. Upon clinical examination, study subjects were grouped into healthy, caries and periodontitis and their saliva was collected. A highly specific point-of-care compatible dual color qPCR assay was developed and used to study the above-mentioned bacteria of interest in the collected saliva. Assay performance was compared to a commercially available microbial reference test. Eight out of ten taxa that were investigated during this study were strong discriminators between the periodontitis and healthy groups: *C. rectus*, *T. forsythia*, *P. gingivalis*, *S. mutans, F. nucleatum*, *T. denticola*, *P. intermedia* and oral *Lactobacilli* (*p* < 0.05). Significant differentiation between the periodontitis and caries group microbiome was only shown for *S. mutans* (*p* < 0.05). A clear distinction between oral health and disease was enabled by the analysis of quantitative qPCR data of target taxa levels in saliva.

## 1. Introduction

Although oral diseases such as caries and periodontitis are less severe in terms of lethality, their health economic burden is substantial. Primary care accounts for around 14% of total health spendings in OECD countries. Within the primary care costs, dental care accounts for up to 40% [1]. According to a global burden of disease study in 2017, oral infections affect almost 3.47 billion people worldwide and are therefore the most prominent chronic infections [2]. For both caries and periodontitis, the oral microbiota is strongly associated with disease stage and progression. Over the past decades there has been a growing body of the literature investigating bacterial profiles of healthy individuals, subjects with periodontitis and dental caries [3,4,5]. The oral health of patients that are suffering from caries is not restored by the eradication of a caries lesion rather a shift in the patient’s oral microbiota. This change can be achieved by an improvement in dietary habits and the establishment of adequate oral hygiene procedures [6]. Tissue destruction of periodontitis patients is characterized by local inflammation triggered by a dysbiosis of naturally occurring oral bacteria. The level of disease can be modified by several risk factors, such as smoking, diabetes [7], genetic factors and age. While both oral diseases differ in their clinical appearance, the microbiota shift to specific pathogens during disease seems a common ground. Prior to treatment, the disease must be diagnosed, and its origin specified by anamnesis and clinical examination in the dental practice. Furthermore, a recall system and ongoing monitoring of patients is recommended to ensure stable conditions or enable re-interventions in an early stage of relapse. Up to now, diagnosis is typically based on surrogate clinical measures, such as bleeding on probing, prior to treatment whereas the microbial factor is rarely taken into consideration for further treatment planning. Knowledge about the patients’ microbiota, however, would simplify effective treatment outcomes and help in adjusting the use of antibiotics [8]. Research activities in the past were mainly focusing on periopathogens of subgingival plaque or single tooth sites. Only recently, has research focused on the quantitative analysis of oral taxa in saliva despite its easy and non-invasive access [9]. Progresses have been made by identifying host-response markers and microbial plaque biofilm levels by quantitative polymerase chain reaction (qPCR) [10], thereby replacing traditional culture-based approaches with novel analytical technologies. Meanwhile several commercial kits are available on the market such as the iai PadoTest, VariOR Dento or MyPerioPath that enable the partial characterization or quantification of patients’ microbiota. Most commercial kits, however, require samples to be shipped to centralized laboratories. Fast analysis on-site is impossible and patients are committed to several appointments again, resulting in substantial costs [9]. Hence, there is great need for near patient testing involving easy to use point-of-care (POC) tests that enable timely diagnosis without the need of highly trained personnel [11].

In this direction, our work consists of two main experimental blocks: (i) the development of a rapid and sensitive POC compatible qPCR assay enabling the detection of ten oral bacteria including amplification controls; and (ii) the collection of a saliva sample cohort, consisting of healthy, periodontitis and caries individuals with the scope of oral health assessment via qPCR.

The present study was designed to collect and analyze a total of 214 saliva samples including healthy donors and patients with caries or periodontitis. Sample material was analyzed via POC compatible qPCR assays, followed by statistical analysis. Assay performance was compared against the commercially available iai PadoTest. Overall, this study has characterized the microbial profile of study participants, identified and differentiated diseased from healthy subjects on a microbial level, and evaluated the assays for further applications.

## 2. Experimental Section

### 2.1. Study Population

Systemically healthy individuals (*n* = 256) visiting the Center of Dental Medicine, Zurich, Switzerland, for their dental treatment were asked to participate in the study. Information on the general health status indicating systemically healthy conditions was checked during anamnesis (diabetes, heart disease, infections such as tuberculosis, hepatitis sexually transmitted diseases, HIV/AIDS, tumor diseases, and gastric digestive disorders causing vomiting were considered exclusion criteria). To be eligible, participants had to be aged at least 18 years, ensure the willingness to comply with the study requirements, and have either caries or periodontitis: (i) caries group: ≥2 open dentinal caries lesions and periodontal screening index (PSI) ≤2 in ≥4 sextants; (ii) periodontitis group: PSI ≥3 in ≥3 sextants and ≤2 open dentinal caries lesions [12]; or (iii) show orally healthy conditions (control group: no caries lesion, PSI scores ≤2 in all sextants). Clinical subgroups were additionally documented within the caries and periodontitis group, as pure caries (no dental pockets, PSI ≤2 in all sextants), caries + (with dental pockets, PSI ≤2 in ≥4 sextants), pure periodontitis (without caries lesions), and periodontitis + (with caries lesions, ≤2 open dentinal caries lesions). Exclusion criteria were: participation in any other clinical trial or active periodontal therapy using surgical interventions within the last three months prior to study enrollment, and heavy smoking (>10 cigarettes a day). Pregnant or lactating subjects were not allowed to participate. Patients taking antibiotics during the study or within the last 6 months were not allowed to participate in order to standardize the systemically healthy state and minimize bias in the bacterial saliva profiling. Out of 256 individuals, 214 were enrolled in the study.

Dentists working at the Center of Dental Medicine, Zurich, Switzerland, were instructed how to check and inform their patients for potential enrollment in the study. Subjects were recruited from September 2018 to April 2019. The study design and protocol were approved by the local ethics Swiss committee (BASEC-no. 2016-00435). All participating subjects signed a written informed consent prior to saliva donation. Saliva samples were coded upon collection and retracing was only applicable for attending dentists using a decoding data sheet to guarantee data privacy.

### 2.2. Study Design

The study was a case-control study involving healthy and orally-diseased patients, with collection of biological material (saliva sampling) taking place at one center (Center of Dental Medicine, Zurich, Switzerland). All subjects were asked to attend two appointments. At the first visit (30–60 min), a routine dental examination took place and subjects were informed about the study details and screened for potential inclusion and exclusion criteria. The clinical grouping into caries, periodontitis, or healthy was performed by the respective dentist. At the second visit, subjects visited the Center of Dental Medicine to donate unstimulated saliva. Upon arriving at the second visit, subjects underwent a coding process, depending on their group, initials, and ascending enrollment number.

### 2.3. Sampling of Saliva

**Figure 1:**
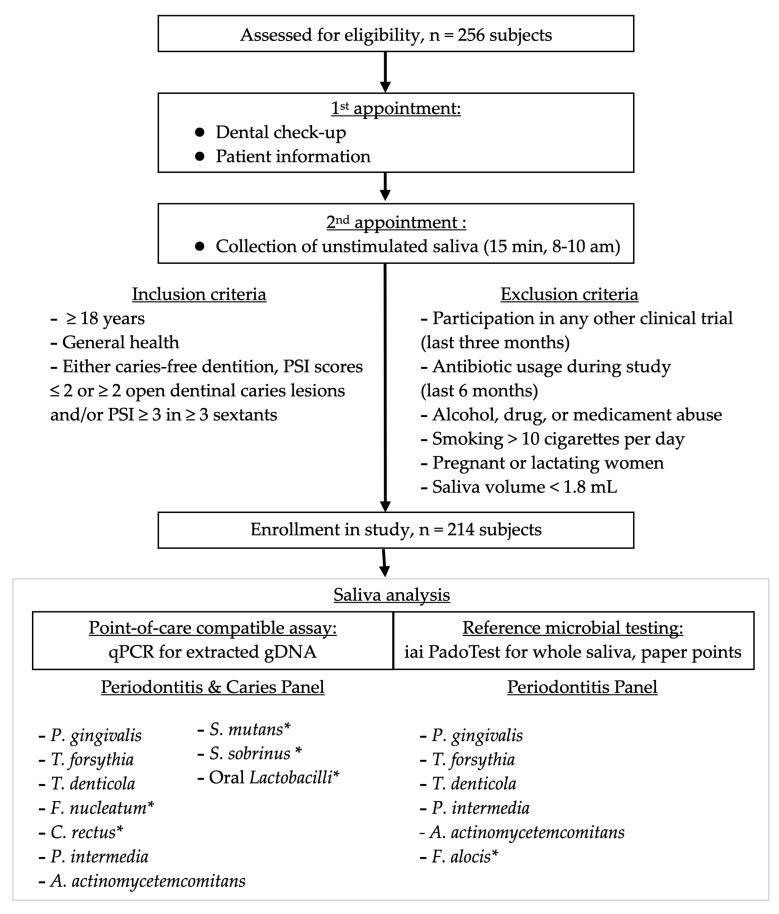
Flow-chart of the study, illustrating recruitment, study population, saliva sampling and saliva analysis. * Differences in bacterial species between the two analysis methods.

Subjects were instructed not to eat the night before the saliva donation and were asked to refrain from all oral hygiene procedures in the morning. Only water consumption was allowed at all time points. All appointments for saliva collection were scheduled between 8.00–10.00 a.m. [13,14]. Saliva donation was performed using a modified collection method based on the guidelines for the collection of unstimulated and stimulated whole saliva [15]. Subjects were instructed on how to donate saliva using a video sequence, explaining and simulating the procedure for standardization. In brief, subjects were asked to swallow at the start, keep the mouth slightly open and let the saliva drain into the test tube. In contrast to the recommendations from Navazesh & Kumar [15], subjects were asked to donate unstimulated saliva for 15 min instead of 5 min. At the end, the remaining saliva was spat into the test tube and the collection was stopped. The test tubes with whole unstimulated saliva were coded, vortexed and aliquoted in DNA low bind tubes (DNA LoBind, Eppendorf, Wesseling-Berzdorf, Germany). Test tubes with less than 1.8 mL in total were discarded (Figure 1). All aliquots were stored at −80 °C until further analysis.

### 2.4. DNA Extraction for qPCR

For the extraction of genomic DNA, the GenElute^TM^ Bacterial Genomic DNA Kit (Sigma-Aldrich, St. Louis, USA) was used with the protocol for Gram-positive bacterial preparation and *Streptococcus* species (addition of 250 units/mL mutanolysin) with a prolonged lysis step. A total of 920 µL of thawed aliquoted whole saliva was spun down at 18,000 rcf for 3 min prior to discarding the supernatant. The pellet was resuspended in the enzyme solution (lysozyme + mutanolysin) and incubated for 1 h at 37 °C on a Thermomixer (1400 rpm, Eppendorf, Wesseling-Berzdorf, Germany). RNase A treatment was performed according to the manufacturer’s instruction. The proteinase K lysis was prolonged from 10 to 30 min at 55 °C and 1400 rpm. The column purification was performed according to the manufacturer’s instruction. The elution was conducted with 135 µL of a 10 mM TrisHCl solution (pH 8.8) and the eluate was stored at −25 °C in the dark until further analysis.

### 2.5. Oligonucleotide Design

De novo primer and probe sets have been designed for *P. gingivalis*, *T. forsythia*, *T. denticola*, *F. nucleatum*, *C. rectus*, *P. intermedia*, *A. actinomycetemcomitans*, *S. mutans*, *S. sobrinus* and orally associated *Lactobacilli* (Table 1). Apart from *S. sobrinus* and *A. actinomycetemcomitans*, all oligonucleotides are targeting the highly conserved 16S ribosomal RNA gene. Due to the high sequence similarity within the *Streptococcus* genera, the 23S rRNA (uracil-5-)-methyltransferase RumA gene was selected for the detection of *S. sobrinus*. The high heterogeneity of available *A. actinomycetemcomitans* 16S rRNA sequences complicated robust assay design and an alternative target was required. The virulence factor and toxin gene LtxA gene [16] is involved in aggressive periodontitis and was chosen for primer and probe design [17,18]. Sequences were extracted from the “Nucleotide” database provided by the National Center for Biotechnology Information (NCBI) as well as the “Human Oral Microbiome Database”(HOMD) of the Forsyth Institute in Cambridge, Massachusetts [19]. Multiple sequence alignment was performed using Clustal Omega [20]. Conserved sequences were visualized via Jalview and subjected to Primer3 for the final oligonucleotide design.

### 2.6. Primer and Probe Specificity

Specificity as well as coverage were first confirmed in silico using Primer Blast in combination with the “not redundant/nucleotide (nr/nt)” database, “chromosomes of all organism” and “HOMD 16S rRNA RefSeq” databases. All sequences passing the in silico specificity and coverage check were cleared for lab analyses. Bacterial genomic reference DNA was ordered at the Leibniz Institute DSMZ—German Collection of Microorganisms Cell Cultures GmbH and the American Type Culture Collection (ATCC) (Table 1). Specificity was confirmed by melting curve analysis and agarose gel electrophoresis following qPCR.

### 2.7. Duplex qPCR

The overall goal was to develop a POC compatible dual color qPCR that can be easily integrated into microfluidic based diagnostic platforms [21]. Point-of-care assay demands, applicable also for this study have been previously defined in work related to POC integration [22]. To reach clinical requirements a quantifiable range of 10 ng to 0.1 pg total bacterial target DNA per qPCR reaction was defined. Bacterial reference DNA (Table 1) was quantified via the NanoDrop 2000 and the Quant-iT™ dsDNA Assay Kit (Thermo Fisher Scientific Inc., Waltham, Massachusetts, USA). DNA was serially diluted and subjected to qPCR. External standard curves for each taxon were generated in three consecutive qPCR runs utilizing six datapoints in 10-fold increments ranging from 10 ng to 0.1 pg (Supplementary Material, Table S1). Detection limits were differing based on the qPCR assay performance of each primer and probe set (Supplementary Material, Figure S1). Each reaction was done in triplicate using a custom “TaqMan Lyophilized 1-Step qPCR MasterMix” (Thermo Fisher Scientific Inc.) with 3.5× master mix. Constituents of this lyophilized pellet shaped reaction mastermix were adapted towards the needs of multicolor qPCR requirements and microfluidic platforms. Enzyme and buffer concentrations have been improved to guarantee optimal qPCR resolution and fluorescence intensities. The lyophilized nature enabled long-term storage at room temperature and the pellet shape fulfills pick and place requirements of manufacturing lines. For this study the pellet volume was downsized to 46 µL due to geometric requirements of miniaturized POC devices. Each qPCR of patient material was run in a dual color format utilizing two TaqMan probes. Oligonucleotide probes that were utilized for the detection of target bacteria were labelled with 6-Carboxyfluorescein (6-FAM). The probe which was specific for the internal control was labelled with Roche’s proprietary LightCycler Red 610 (Table 1).

The amplification control consisted of 0.01 ng *Serinicoccus marinus* genomic DNA, which was spiked into each reaction. Following extensive literature and in silico studies, this strain was chosen due to its absence from the human oral microbiome or consumed food. Following the finalization of standard curves, qPCR reaction mixes for all 214 patient qPCR runs were prepared within one day including positive controls of reference bacterial DNA, internal standard DNA, negative controls, as well as primers and probes. One aliquot per patient was stored at −80 °C to minimize variation due to repetitive freeze–thaw cycles. Subsequently, qPCR was performed on a Roche Light Cycler 480II. A two-step cycling protocol was used starting with an initial activation at 95 °C for 2 min followed by 40 cycles at 95 °C for 3 sec and 60 °C for 30 sec. Inconclusive amplification curves that were labelled with “detector call uncertain”, “early or late Cq call” were not further subjected to analysis. Cycle quantification values (Cq) within the previously determined standard curves were automatically determined by the “Second Derivative Maximum Method” [23]. Data below the detection limit were set to zero (Supplementary Material, Table S1).

Bacterial quantity was determined with the previously established standard curves and rounded down to whole numbers.

### 2.8. Conversion into Genome Equivalent

The genome size for each reference strain was extracted from NCBI Assembly and multiplied by the average molecular weight of a base pair. The derived total genome mass was then used to calculate the number of genomes from the measured DNA weight, which was defined as one genome equivalent (ge). All masses including the established standard curves of 10 ng–0.1 pg as well as qPCR data of patients were converted into genomic equivalents and used for further analysis.

### 2.9. Reference Microbial Testing

The aliquoted whole saliva samples were subjected to a commercially available molecular diagnostic test for the identification of periodontal pathogens (iai PadoTest, Institut für Angewandte Immunologie IAI AG, Zuchwil, Switzerland), which served as a reference to the POC compatible qPCR assay. The test provides a quantitative analysis for total bacterial loads of *A. actinomycetemcomitans, F. alocis*, *P. intermedia* of the “orange complex” and all bacterial species of the “red complex”: *P. gingivalis*, *T. forsythia*, and *T. denticola* [24]. The original sampling procedure was modified towards saliva processing. Instead of applying paper points in dental pockets for 10–15 s, 4 paper points (Roeko Iso 55, Coltène, Altstätten, Switzerland) were immersed in 40 µL of thawed whole saliva in a tube. The tube with the paper points was sent to the Institut für Angewandte Immunologie IAI AG for microbial analysis (iai PadoTest). The multiplex real-time qPCR assay estimates bacterial cell counts on the basis of 16S rRNA [25].

### 2.10. Statistics

For the dataset including only measurements above the detection limit, median values and interquartile ranges for bacterial loads of each species (in ge/mL) were calculated for each clinically defined group (caries, periodontitis and healthy) and for both, the newly-developed qPCR and the commercially available iai PadoTest. Within each bacterial strain, the bacterial loads of the three clinical groups were then statistically compared against each other using Kruskal–Wallis rank sum tests followed by pairwise comparisons using Conover’s-test. The *p*-values were adjusted for multiple testing according to Holm and the significance level was set to α = 0.05.

In order to compare both assessment methods’ sensitivity, measurements below detection limit were tentatively replaced by zero values (ge/mL). Using this larger dataset, the measurements of the POC compatible assay were graphically compared to those of the iai PadoTest for the following taxa: *P. gingivalis*, *T. forsythia*, *T. denticola*, *P. intermedia*. All statistical analyses and plots were computed with the statistical software R [26], including the packages ggplot2 [27] and PMCMRplus [28].

## 3. Results

### 3.1. Study Population

**Figure 2:**
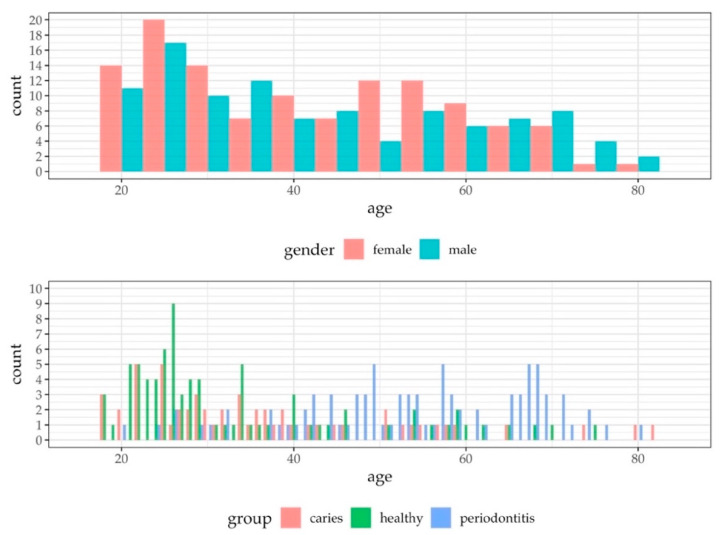
Histograms presenting the age distribution by gender and by group (healthy, caries and periodontitis) for all study participants. While gender is almost equally distributed across all age groups, healthy individuals are more frequently found in younger age groups, caries patients in younger to middle-aged groups, and periodontitis patients in the middle-aged and elderly study population.

The study population is presented in Figure 1. A total of 256 volunteers, aged 18–82 (mean 43 years, 131 females) participated in the study after subjects were screened for inclusion/exclusion criteria. The numbers and reasons for sample exclusions were as follows: six due to insufficient saliva volumes (<1.8 mL); 34 due to an ambiguous pathological clinical phenotype; and two due to volunteer ages (<18 years). Overall, 214 saliva samples were subjected to the final analysis. Due to the explorative nature of this study, gender was not stratified, however, gender was almost evenly distributed over all ages (Figure 2). The age distribution differed slightly between all groups (Figure 2). Younger subjects frequently belonged to the healthy group (mean: 34 years) and younger to middle-aged subjects to the caries group (mean: 38 years). Patients suffering from periodontitis were mainly found in the middle-aged and elderly population (mean: 54 years).

### 3.2. Characteristics of the qPCR Data

**Figure 3:**
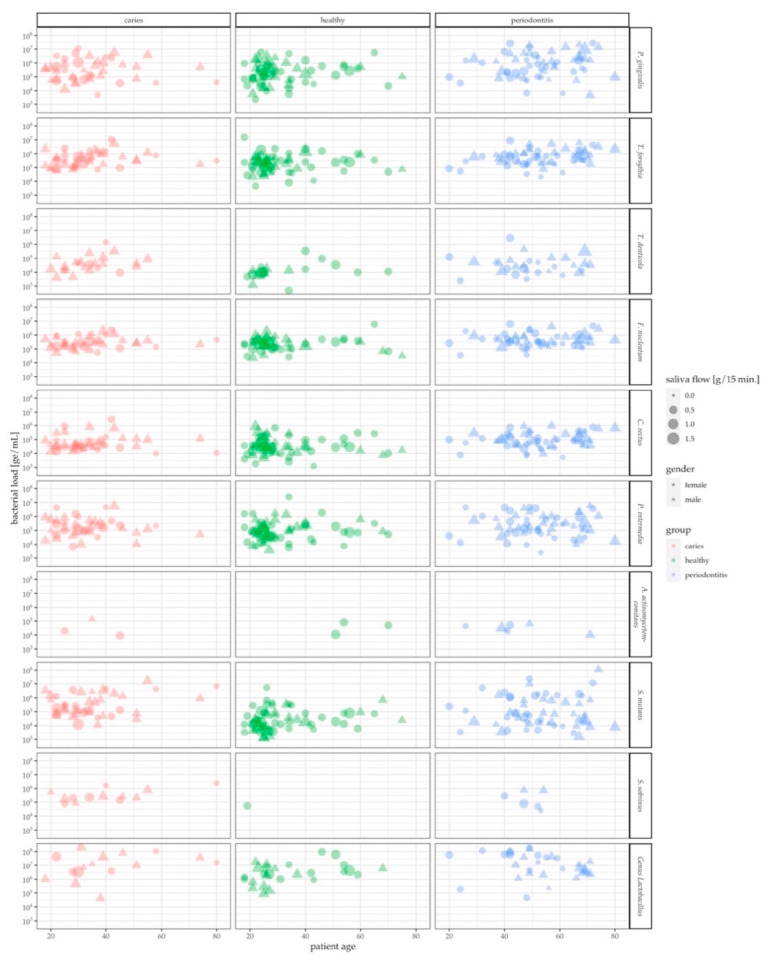
Overview of the age, gender and saliva flow rate distribution. Depending on their classification, data from caries patients are displayed in red (left), healthy individuals in green (middle) and periodontitis patients in blue (right). The bacterial loads in genome equivalents (ge/mL) are presented for all analyzed bacterial strains, captioned for each strain on the right.

Figure 3 depicts the distribution of parameters collected in the context of this study such as age, gender, health condition, genome equivalents of target species and saliva flow in g/min. It appears that there is hardly any detrimental bias in age or gender among the three study groups. Overall, caries patients tended to be younger than periodontitis patients. The largest clusters, generally, could be observed in the healthy population of people below 30 years in age. It appears that females exceeded males in the healthy study group that are 40 years and older. The number of male periodontitis patients, however, is higher than the one of females in the age group of 60 years and above. Saliva flow altogether was evenly dispersed across health conditions and age. Only a few patients were positive for *A. actinomycetemcomitans* and *S. sobrinus*, often associated with very low levels of target genome equivalents. Very high bacterial levels could be observed for *P. gingivalis*, *S. mutans* and oral *Lactobacilli*, particularly in the periodontitis group (Table 2).

### 3.3. Assessment of Oral Health Status by Pathogen Levels in Saliva

**Figure 4:**
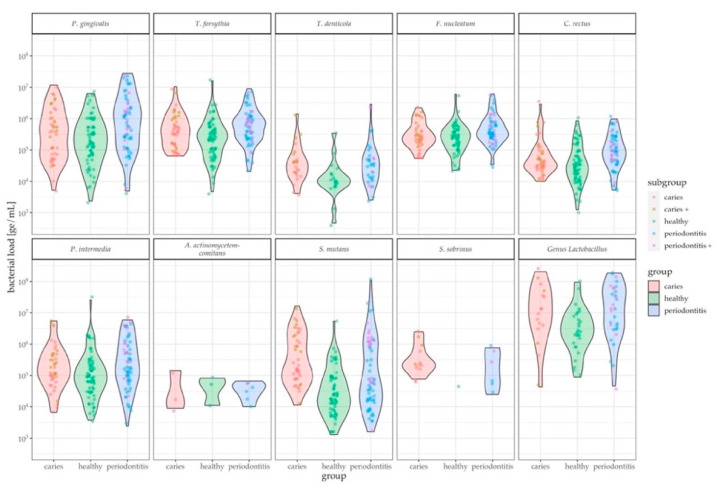
Quantity of each bacterial strain in saliva, in genome equivalents per mL (ge/mL), detected by the POC compatible qPCR assays for each study group. Counts within the violins are colored based on corresponding clinical subgroups (within the caries violin: yellow = caries patients with periodontitis, red = pure caries patients; within periodontitis violin: violet = periodontitis patients with caries, blue = pure periodontitis patients; healthy patients do not have subgroups, all counts in green. In general, healthy subjects can be statistically distinguished from periodontitis patients for all targets except *A. actinomycetemcomitans* and *S. sobrinus*, the most prominent being *P. gingivalis*, *T. forsythia* and *C. rectus*. A difference between caries and periodontitis groups can only be seen for *S. mutans*.

The tested taxa are associated with caries and periodontitis. While there are tests for gingival crevicular fluid (GCF), saliva testing is not commonly performed. In the current study it was shown that salivary pathogen levels can discriminate between healthy and disease states, which was tested by a pairwise comparison between the oral infection groups and the control group (healthy-periodontitis, healthy-caries). Differences were found mainly between the control and periodontitis groups: highly significant differences in species load were detected in *C. rectus, T. forsythia,* and *P. gingivalis* (*p*-value ≤ 0.001), followed by *S. mutans* and *F. nucleatum* (*p*-value = 0.05–0.001), as well as *T. denticola*, *P. intermedia*, and oral *Lactobacilli* (*p*-value = 0.01–0.05). Significant differences between the healthy and caries groups were only obtained by *S. mutans* (*p*-value = 0.05–0.001). In addition, *S. mutans* can discriminate between caries versus periodontitis (*p*-value = 0.05–0.001) (Figure 4, Table 2).

**Figure 5:**
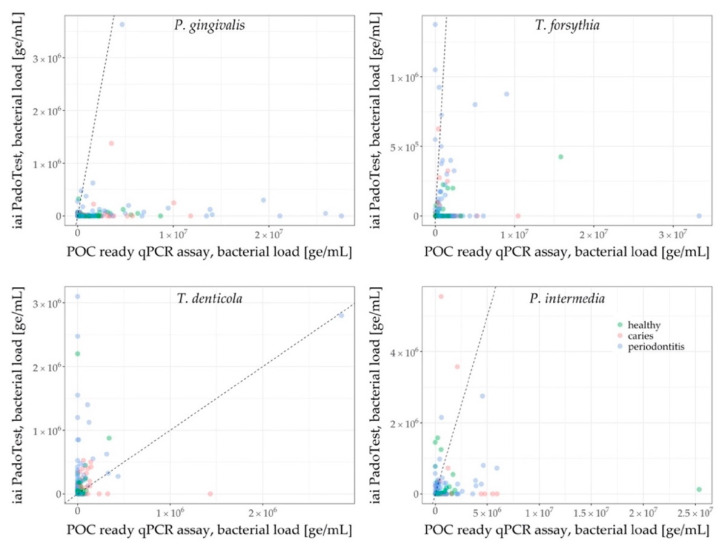
qPCR results for *P. gingivalis*, *T. forsythia*, *T. denticola* and *P. intermedia.* Both the iai PadoTest and the POC compatible qPCR assay results were converted into genome equivalents. Groups are color-coded (red: caries, green: healthy, blue: periodontitis). The comparison shows much higher sensitivity of the POC compatible qPCR assay in saliva, compared to the iai PadoTest for *P. gingivalis* and *T. forsythia* (and less pronounced for *P. intermedia*). Only *T. denticola* in turn, is detected more frequently using the iai PadoTest than the POC compatible qPCR assay.

qPCR results for *P. gingivalis*, *T. forsythia*, *T. denticola* and *P. intermedia* of both the commercial iai PadoTest and the newly-developed POC compatible qPCR assay were converted into genome equivalents and are shown in Figure 5. Sensitivity of the qPCR assays were superior to the iai PadoTest, for *P. gingivalis*, *T. forsythia*, and *P. intermedia*. It was shown that lower pathogen levels, especially samples originating from the caries and periodontitis groups, were more likely to be detected with the assay that was designed specifically for this study. Only a minority of samples was solely detected by the iai PadoTest, especially for *T. denticola*. The higher resolution of the POC compatible qPCR enabled a more comprehensive overview of the patients’ microbiome.

## 4. Discussion

Much of the previously conducted research has focused on the qualitative identification of species in gingival crevicular fluid (GCF) and microbial plaque biofilms [10,29,30]. Quantitative approaches, such as qPCR assays, have been successfully utilized in saliva diagnostics in a periodontitis cohort [31] and healthy adults [32]. However, there is currently a lack of studies quantifying and analyzing the presence of oral taxa in large and clearly distinguished study groups. Hence, the primary goal of this study was the development of a diagnostic POC compatible qPCR assay that can be used to identify patients who are potentially prone to caries or periodontitis and to maintain their oral health. The highly specific and sensitive qPCR assay developed within this study is intended for POC applications and was pre-validated with collected clinical samples. Point-of-care compatibility was achieved by selecting assay components which were chosen based on their performance in low volumes and the ability to be lyophilized, thereby ensuring long term stability, ease of manufacturing and integration into existing POC platforms [33]. Furthermore, the development of a novel, openly accessible amplification control based on genomic DNA of the Gram-positive bacterium *S. marinus* [34] was achieved. Finally, the de novo design and validation of oligonucleotides and optimization of fluorophores as well as the refinement of two-step qPCR cycling protocols resulted in a robust POC compatible assay.

The performance of the POC compatible qPCR assay was compared against the commercially available iai PadoTest by analyzing qPCR results of bacterial pathogens such as for *P. gingivalis*, *P. intermedia*, *T. denticola* and *T. forsythia*. This study identified and confirmed key pathogens in saliva, which were associated with oral diseases [35,36,37], that can distinguish microbial profiles of each group (healthy, periodontitis, caries). The qPCR data showed that *P. gingivalis* was very prominent in most periodontitis samples, followed by *T. forsythia*, *C. rectus*, *F. nucleatum*, *S. mutans*, *T. denticola*, and members of the genus *Lactobacillus*. Interestingly, *A. actinomycetemcomitans* levels did not differ significantly between the groups. This lack of microbial differentiation by *A. actinomycetemcomitans* between groups has been also described in other studies, detecting periodontal pathogens in saliva samples of differently diseased populations [31,38]. Possible reasons might be that the *A. actinomycetemcomitans* carrier can differ in the clinical outcome of the disease. The JP2 clone, for example, is common in specific populations or the pathogenesis of aggressive periodontitis [39]. Additional reasons may also be based on its higher prevalence among younger subjects with periodontitis and its overall detection in one third of healthy adults [39,40]. This age-related prevalence could explain the lack of group differentiation by *A. actinomycetemcomitans* throughout the study, since age distribution differed between the groups: the healthy group consisted mainly of a younger population (mean: 34 years), while middle-aged and elderly patients were more often grouped to periodontitis (mean: 54 years). *S. mutans* was the key bacterium [41] detected in samples from patients with a diagnosis of dental caries, followed by *T. denticola*. Statistical differentiation between both oral infection groups was, however, only possible for *S. mutans* with the POC compatible qPCR assay, and *P. gingivalis* and *T. denticola* with the iai PadoTest. Sensitivity of the newly-developed qPCR assay was superior to that of the iai PadoTest (Figure 5), and the detected panel of the new qPCR assay was also broader. These features enabled a more comprehensive overview of the patients’ microbial profile. It must be noted, however, that the iai PadoTest was developed for the analysis of dental plaque collected from gingival crevices or periodontal pockets; on the other hand, we used saliva as a test sample matrix, which is an important feature of our study as saliva is a key diagnostic sample matrix, given its non-invasive collection method. Due to project-dependent limited recruitment duration and the overall explorative nature of this study, a rather broad clinical grouping was applied. The periodontitis group therefore consisted of patients displaying PSI code 3 and 4, which is indicative of mild/moderate or advanced periodontitis according to Preshaw [42]. The fact that both oral disease groups consisted of mixed patients (evident caries or periodontitis patients and patients with both diseases to different extents) might explain the bimodalities as shown in Figure 4. Several violin plots illustrate two peaks of patients within the caries group (*P. gingivalis*, *S. mutans*) and within the periodontitis group (*P. gingivalis*, *T. forsythia*, *S. mutans* and the genus *Lactobacillus*). The peaks might indicate different clinical subgroups. Differences in the microbial population of groups presenting the same disease, however, were not significant. While some patients with varying degrees of caries lesions can show a high periodontal resistance and vice versa, some periodontitis patients do not show any sign of caries lesions, and many patients present themselves at the dental clinic with signs of both diseases. The differentiation into healthy, caries and/or periodontitis appears rather difficult in these cases and after careful consideration an individual decision for diagnosis must be made.

Overall, these newly developed qPCR assays were able to differentiate the oral health status of the patients, according to the analysis of the selected microbial taxa in their saliva. The strongest differences in bacterial targets were found between healthy individuals and periodontitis patients (*C. rectus, T. forsythia, P. gingivalis* (*p*-value ≤ 0.001), *S. mutans, F. nucleatum* (*p*-value = 0.05–0.001), *T. denticola*, *P. intermedia*, oral *Lactobacilli* (*p*-value = 0.01–0.05). Significant differences were found between healthy and caries patients, as well as joint caries and periodontitis patients by *S. mutans* (*p*-value = 0.05–0.001).

## 5. Conclusions

This study showed the high performance and quality of the newly-developed POC compatible qPCR assays in clinical samples. This offers a high potential for future applicability on personalized monitoring and treatment based on the patient oral microbiota enabling early intervention and prevention of disease. Furthermore, the nature of the qPCR assays design enables them to be integrated into fully-integrated systems for future implementation at the point-of-care.

## Figures and Tables

**Table 1:** Representation of designed oligonucleotide sets used for the detection of target bacteria. The table includes primer and probe sequences, corresponding fluorescent labels as well as utilized quenchers. 6-FAM = 6-Carboxyfluorescein; BHQ-1 = Black Hole Quencher^®^.

Organism	DNA Sequence (5′-3′)	Strand on Template	Modification at 5′	Modification at 3′	Purification	Reference Material
*Porphyromonas gingivalis*	TTGCTAAGGTTGATGGCGAC	+			Desalted	DSMZ 20709
	ACAAGTGTATGCGGTTTTAGTCC	-			Desalted	
	CGCGTATGCAACTTGCCTTA	+	6-FAM	BHQ-1	HPLC	
*Treponema denticola*	ATTGGGACTGAGATACGGCC	+			Desalted	DSMZ 14222
	AGAAGCATTCCCTCTTCTTCTT	-			Desalted	
	CCGTGTGAATGAAGAAGGCC	+	6-FAM	BHQ-1	HPLC	
*Tannerella forsythia*	TGTACCTTGTGAATAAGCATCGG	+			Desalted	ATCC 43037
	CGGACTTAACAGCCCACCTA	-			Desalted	
	CGGTAATACGGAGGATGCGA	+	6-FAM	BHQ-1	HPLC	
*Fusobacterium nucleatum*	AACTTCGATTTGGGTGGCG	+			Desalted	DSMZ 15643
	AGCTTTCATAATTCTAGGATGCCC	-			Desalted	
	CCTCACAGCTAGGGACAACA	+	6-FAM	BHQ-1	HPLC	
*Prevotella intermedia*	GAACTGGCGGACTTGAGTG	+			Desalted	DSMZ 20706
	AGTAACACTCCCGTACGCTG	-			Desalted	
	CGGAATTCATGGTGTAGCGG	+	6-FAM	BHQ-1	HPLC	
*Campylobacter rectus*	AGCAAATCTATAAAATACGTCCCAGT	+			Desalted	DSMZ 3260
	CCGGTTTGGTATTTGGGCTTC	-			Desalted	
	TACGTTCCCGGGTCTTGTAC	+	6-FAM	BHQ-1	HPLC	
*Aggregatibacter actinomycetemcomitans*	GCTGATACTGCAACGAAAGC	+			Desalted	DSMZ 8324
	CAAGCATTCTCGCACGATCA	-			Desalted	
	CGGGGCTTTCTACTACGGGA	+	6-FAM	BHQ-1	HPLC	
*Streptococcus mutans*	CCAGAAAGGGACGGCTAACT	+			Desalted	DSMZ 20523
	GCCTTTTACTCCAGACTTTCCT	-			Desalted	
	TATTGGGCGTAAAGGGAGCG	+	6-FAM	BHQ-1	HPLC	
*Streptococcus sobrinus*	CCAAAATTCCGCAGAGTCGC	+			Desalted	DSMZ 20742
	CCTTCAAAGCACCAGGGACA	-			Desalted	
	TGCAGGTCAAACAACGGATTCC	+	6-FAM	BHQ-1	HPLC	
*Oral Lactobacilli*	GTGCAGAAGAGGASAGTGGA	+			Desalted	DSMZ 4905
	ATCCTGTTCGCTACCCATGC	-			Desalted	
	ATGGAAGAACACCAGTGGCG	+	6-FAM	BHQ-1	HPLC	
*Serinicoccus marinus*	CGCAGAGATGTGGTTTCCCT	+			Desalted	DSMZ 15273
	TCCCATGAGTCCCCAACCA	-			Desalted	
	AGCGCAACCCTCGTTCCATGT	+	LightCycler Red 610	BHQ-1	HPLC	

**Table 2:** Median values of bacterial loads (in ge/mL) and interquartile ranges (IQR) of all bacterial strains measured by the POC compatible qPCR assay and iai PadoTest, separated into groups. Statistically significant differences in group comparisons (healthy versus caries, healthy versus periodontitis, caries versus periodontitis) are marked with stars; * 0.05 > *p* ≥ 0.01, ** 0.01 > *p* ≥ 0.001, *** *p* < 0.001).

		Healthy		Caries		Periodontitis		*p*-Value		
Panel	Strain	Median	IQR	Median	IQR	Median	IQR	Healthy-Caries	Healthy-Periodontitis	Caries-Periodontitis
POC compatible qPCR Assay	*P. gingivalis*	193,889	537,489	334,152	1,007,053	948,848	2,519,962	0.232	<0.001 ***	0.064
	*T. forsythia*	244,480	404,683	360,430	806,900	635,674	839,909	0.053	<0.001 ***	0.154
	*T. denticola*	11,025	9815	36,482	44,387	30,683	51,061	0.007 **	0.011 *	0.638
	*F. nucleatum*	239,745	316,713	252,436	321,205	366,993	541,370	0.319	0.004 **	0.081
	*C. rectus*	29,311	64,630	41,116	60,922	82,206	195,432	0.068	<0.001 ***	0.059
	*P. intermedia*	83,001	192,885	146,802	376,529	185,607	989,823	0.107	0.012 *	0.472
	*A. actinomycetemcomitans*	49,966	35,481	20,173	67,154	37,932	28,284	1.000	1.000	1.000
	*S. mutans*	22,218	72,897	194,821	1,305,829	68,350	871,276	<0.001 ***	0.004 **	0.004 **
	*S. sobrinus*	55,417	-	231,463	380,989	185,138	569,481	0.510	0.680	0.680
	Genus *Lactobacilli*	2,478,206	6,090,513	11,086,039	38,167,335	11,247,675	53,562,778	0.065	0.021*	0.894
iai PadoTest	*A. actinomycetemcomitans*	-	-	-	-	-	-	-	-	-
	*T. forsythia*	87,500	150,000	450,000	175,000	300,000	525,000	0.140	0.050	0.510
	*P. gingivalis*	50,000	25,000	25,000	12,500	75,000	112,500	0.235	0.011 *	0.011 *
	*T. denticola*	125,000	150,000	100,000	93,750	200,000	362,500	0.432	0.130	0.016 *
	*P. intermedia*	162,500	556,250	125,000	287,500	275,000	206,250	0.820	0.820	0.400
	*F. alocis*	75,000	150,000	75,000	118,750	100,000	275,000	1.000	1.000	1.000
	Universal Primer	671,500,000	1,136,250,000	873,000,000	1,317,000,000	744,000,000	1,055,500,000	0.350	0.720	0.720

## Supplementary Materials

The following are available online at https://www.mdpi.com/2077-0383/9/9/2945/s1, Figure S1. Duplex qPCR results of three consecutive PCR runs for all oral targets. Table S1: Data (triplets) of standard curves for all bacterial taxa; NC = negative control.
