# Supplementary material for: Microbial Analysis of Saliva to Identify Oral Diseases Using a Point-of-Care Compatible qPCR Assay
Source: J Clin Med. 2020 Sep 11;9(9):2945. doi: 10.3390/jcm9092945 (PMC7565348; doi:10.3390/jcm9092945)
Supplement: Supplementary file 1 [file jcm-09-02945-s001.pdf]

Primer Efficiency

A quantifiable range between 10 ng–0.1 pg total DNA per reaction was aimed for point-of-care (POC) platforms. Therefore, duplex qPCR reactions were set up always including a 10-fold dilution series for one caries or periodontitis target (FAM) and 0.01 ng *Serinicoccus marinus* (LC610) to meet POC requirements. qPCR for reference material was repeated three times, each time preparing all reagents and standards from scratch. For a better understanding, oral specialist demanded the display of genome equivalents per ml instead of template per reaction. Each x axis displays the log10 of cells that was calculated on the basis of complete NCBI assembly genomes.

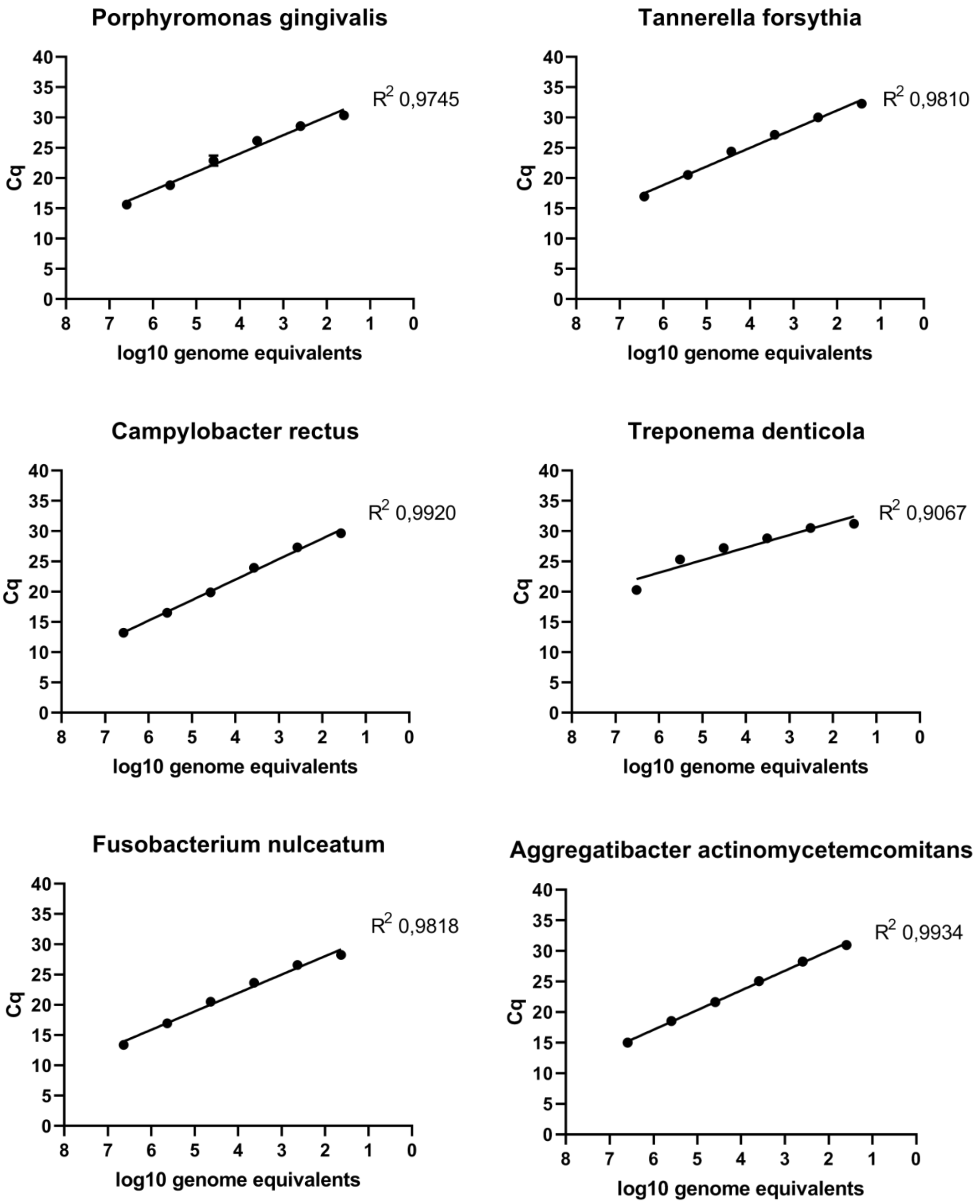

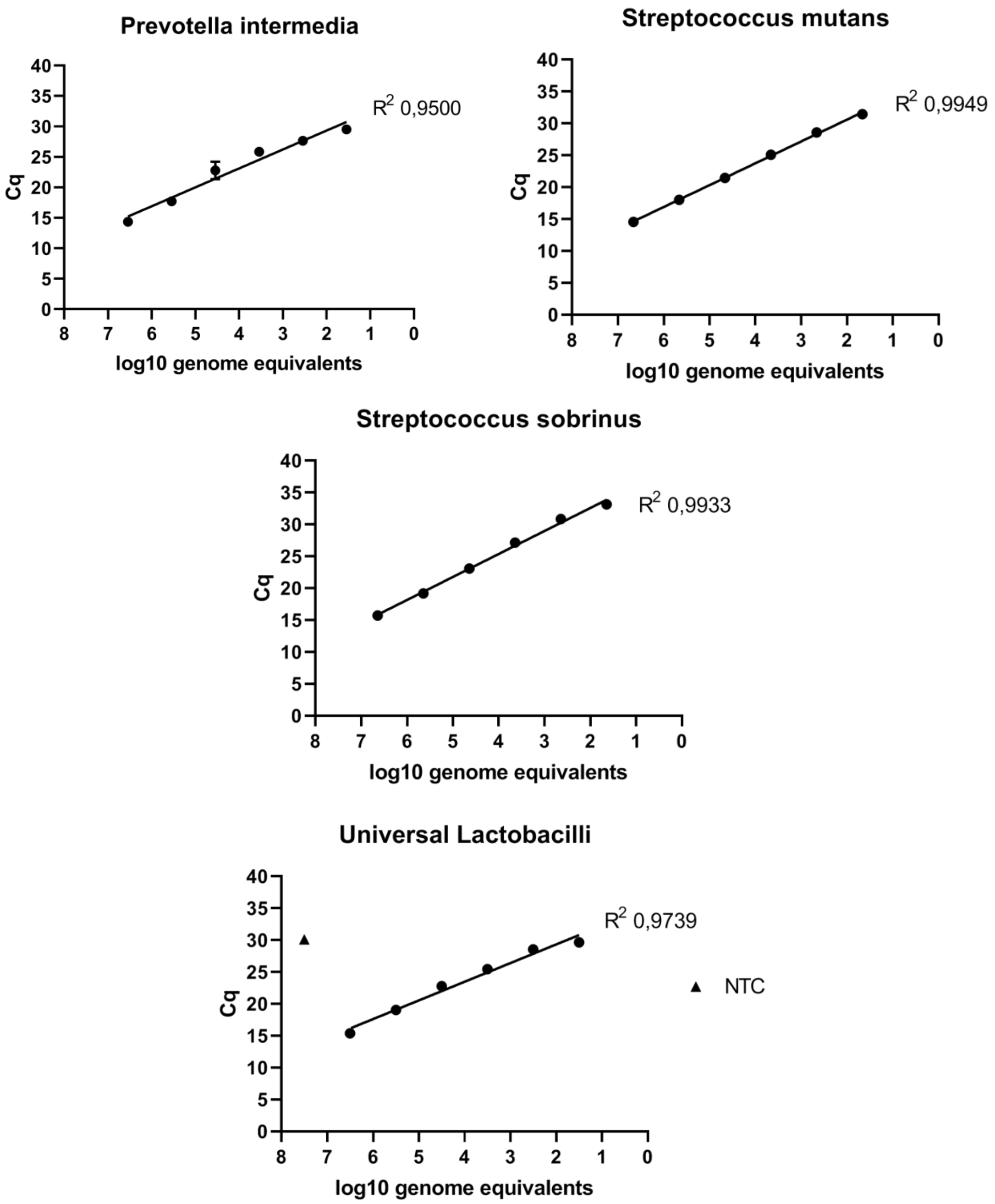

Figure S1. Duplex qPCR results of three consecutive PCR runs for all oral targets.

Table 1. Data (triplets) of standard curves for all bacterial taxa

		**log 10 (CFU/mL)**	**run 1**			**run 2**			**run 3**		
P. gingivalis	NC										
	10ng	6.6	16.44	15.78	15.41	16.48	15.68	15.78	15.01	14.88	14.94
	1ng	5.6	19.06	19.12	19.09	19.2	18.79	19.21	18.39	18.42	18.09
	0.1ng	4.6	23.46	23.8	23.05	23.73	23.29	23.11	21.61	21.97	21.82
	0.01ng	3.6	25.97	26.78	26.48	26.76	26.31	26.62	25.51	25.44	25.33
	1pg	2.6	28.69	28.17	28.34	29.17	28.91	28.46	28.78	28.5	28.29
	0.1pg	1.6	29.95	30.03	30.1	30.19	30.75	30.46	30.55	30.68	30.55
	*0.01pg***	*0.6**	*31.54**	*27.65**	*31.34**	*32.4**	*31.65**	*32.03**	*32.42**	*32.62**	*32.06**
T. forsythia	NC										
	10ng	6.43	17.44	17.77	17.83	17.23	17.05	16.97	15.97	15.98	16.24
	1ng	5.43	20.92	21.1	21.34	20.94	20.68	20.68	19.57	19.67	19.62
	0.1ng	4.43	24.98	24.93	25.13	24.94	24.63	24.59	23.38	23.34	23.41
	0.01ng	3.43		27.4	27.13	27.13	27.9	27.49	26.7	26.87	26.66
	1pg	2.43		30.29	29.77	30.29	30.55	29.88	29.67	29.78	29.94
	0.1pg	1.43	31.64	32.24	31.9	32.07	32.37	32.74	32.33	32.9	32.24
	*0.01pg***	*0.43**	*33.09**	*27.31**							
T. denticola	NC										
	10ng	6.51	20.25	20.7	20.4	19.9	20.1	20.32	20.4	20.63	19.93
	1ng	5.51	25.61	25.14	25.63	25.83	24.96	25.26	25.12	25.18	25.13
	0.1ng	4.51	27.04	27.17	27.16	27.13	27.14	27.27	27.29	27.31	27.43
	0.01ng	3.51	28.8	28.81	28.69	28.63	28.89	28.78	28.98	29.01	28.79

	1pg	2.51	30.43	30.52	30.43	30.54	30.61	30.51	30.49	30.75	30.52
	0.1pg	1.51		31.02	31.37	31.38	31.61	30.69	31.38	31.43	30.87
	*0.01pg***	*0.51**									
F. nucleatum	NC										
	10ng	6.63	13.79	13.61	13.83	12.97	13.17	13.57	13.57	12.84	13.06
	1ng	5.63	17.36	17.57	17.51	17.33	17.13	17.02	16.46	16.53	15.62
	0.1ng	4.63	20.96	21.62	20.83	20.67	21.02	20.32	19.77	19.89	19.58
	0.01ng	3.63	23.65	24.06	23.81	23.71	24.04	23.65	23.08	23.44	23.28
	1pg	2.63	26.29	26.57	26.46	26.59	26.85	26.82	26.34	26.74	26.55
	0.1pg	1.63	27.99	28.17	27.95	27.79	28.65	28.31	28.57	28.64	28.27
	*0.01pg***	*0.63**	*29.08**	*28.81**	*29.22**	*29.47**	*29.79**	*29.84**	*29.97**	*29.92**	*0**
C. rectus	NTC										
	10ng	6.57	13.87	13.76	12.52	13.14	13.12	12.34	13.15	13.34	13.66
	1ng	5.57	16.65	16.37	15.92	16.37	16.35	16	16.9	17.24	16.82
	0.1ng	4.57	19.31	19.94	20.26	19.42	19.46	19.67	20.5	19.7	20.57
	0.01ng	3.57	23.83	23.93	23.83	23.89	23.9	23.94	23.88	24.18	24.04
	1pg	2.57	27.55	27.71	27.34	27.09	27.43	27.33	27.08	27.13	27.18
	0.1pg	1.57	29.89	29.84	29.91	29.17	29.44	29.16	29.85	29.84	29.67
	*0.01pg***	*0.57**	*31.73**	*32.69**	*31.56**	*31.09**	*30.76**		*32.17**	*31.65**	
P. intermedia	NC										
	10ng	6.54	14.53	14.84	14.94	13.81	13.94	14.18	13.74	14.12	15.04
	1ng	5.54	18.31	18.29	18.27	17.08	16.97	17.53	17.34	17.94	17.72
	0.1ng	4.54	23.44	24.51	25.08	21.19	21.5	21.2	21.94	22.71	23.59
	0.01ng	3.54	26.42	26.53	26.55	25.61	25.19	25.46	25.52	25.62	25.66
	1pg	2.54	27.76	28.18	27.9	27.51	27.54	27.12	27.67	27.52	27.55

	0.1pg	1.54	29.85	29.96	29.7	29.55	29.27	29.18	29.48	29.21	29.55
	0.01pg**	0.54*	31.3*	30.87*	31.14*				30.49*		30.23*
**A. actinomycetemcomitans**	NC										
	10ng	6.59	14.58	15.06	15.14	14.68	15.63	15.2	14.46	15.34	14.98
	1ng	5.59	18.22	18.6	18.63	17.71	19.21	19.33	17.95	18.77	18.38
	0.1ng	4.59	21.57	21.98	22	21.44	22.02	22.2	21.34	21.14	21.21
	0.01ng	3.59	25.52	25.14	25.13	24.81	24.93	25.18	24.9	25.06	25.22
	1pg	2.59	29.56	28.04	27.77	28.16	27.78	28.02	28.5	28.37	28.38
	0.1pg	1.59	30.81	30.55	30.77	30.85	30.68	30.99	31.05	31.56	31.6
	0.01pg**	0.59*		33.18*	34.34*	34.49*					
**S. mutans**	NC										
	10ng	6.66	14.81	14.71	14.46	14.44	14	14.28	14.58	14.77	14.77
	1ng	5.66	18.24	18.35	18.12	17.91	18.15	17.12	18.08	18.47	17.83
	0.1ng	4.66	21.65	21.66	21.15	21.19	21.66	20.61	22.08	21.75	21.37
	0.01ng	3.66	25.17	25.28	24.76	24.9	24.89	24.72	25.31	25.39	25.42
	1pg	2.66	28.23	28.91	28.17	28.04	28.47	27.98	29.19	29.46	28.82
	0.1pg	1.66	30.83	31.51	30.89	31.13	31.7	31.74	31.82	32	31.4
	0.01pg**	0.66*					33.67*	33.42*			
**S. sobrinus**	NC										
	10ng	6.64	15.89	15.71	15.69	15.99	15.56	15.58	15.81	15.6	15.68
	1ng	5.64	19.01	19.54	18.94	19.5	19.02	19.08	19.24	19.32	19.04
	0.1ng	4.64	23.01	23.08	22.93	22.96	22.96	23.18	23.24	23.15	23.25
	0.01ng	3.64	27.17	27.37	27.16	27.09	26.97	26.92	27.22	27.5	27.06
	1pg	2.64	31.04	31.03	31.01	30.59	30.46	30.4	31.18	30.93	31.05
	0.1pg	1.64	33.39	33.24	33.02	32.85	32.62	32.61	33.34	33.46	33.75

	*0.01pg***	*0.64**									
**Universal *Lactobaccilli***	NC		30.47	30.45	30.12	29.9	29.97	29.94	30.03	30.08	30.02
	10ng	6.5	15.5	15.57	15.09	15.77	15.44	14.78	15.5	15.69	15.01
	1ng	5.5	19.23	18.64	18.52	19.18	19.25	18.43	19.42	19.94	18.87
	0.1ng	4.5	23.03	22.89	21.95	22.2	23.45	22.35	23.01	23.45	22.65
	0.01ng	3.5	25.36	25.54	25.47	25.47	25.45	25.23	25.53	25.64	25.36
	1pg	2.5	28.68	29.05	28.76	28.63	28.7	28.54	27.99	28.56	27.99
	0.1pg	1.5	29.87	30.03	29.96	29.75	29.48	29.45	29.47	29.55	29.15
	*0.01pg***	*0.5**	*29.86**	*29.79**	*29.82**				*29.29**	*29.58**	*0**

NC = negative control.
